# Bezafibrate improves insulin resistance evaluated using the glucose clamp technique in patients with type 2 diabetes mellitus: a small-scale clinical study

**DOI:** 10.1186/1758-5996-6-113

**Published:** 2014-10-17

**Authors:** Hideki Shiochi, Tsuyoshi Ohkura, Yohei Fujioka, Keisuke Sumi, Naoya Yamamoto, Risa Nakanishi, Kazuhiko Matsuzawa, Schoichiro Izawa, Hiroko Ohkura, Kazuoki Inoue, Etsuko Ueta, Masahiko Kato, Shin-ichi Taniguchi, Kazuhiro Yamamoto

**Affiliations:** Division of Cardiovascular Medicine, Endocrinology and Metabolism, Department of Molecular Medicine and Therapeutics, Tottori University Faculty of Medicine, Nishi-chou 36-1, Yonago, Tottori, 683-8504 Japan; Department of Regional Medicine, Tottori University Faculty of Medicine, Yonago, Tottori, 683-8504 Japan; School of Health Science, Tottori University Faculty of Medicine, Yonago, Tottori, 683-8504 Japan

**Keywords:** Bezafibrate, Type 2 diabetes mellitus, Glucose clamp, Meal tolerance test, Japanese patients, Insulin resistance

## Abstract

**Background:**

Bezafibrate is mainly used to treat hypertriglyceridemia. Studies have reported that bezafibrate also improves type 2 diabetes mellitus, but the mechanism has not been fully elucidated. We performed euglycemic hyperinsulinemic clamps (glucose clamp) and meal tolerance tests (MTT) to examine the effects of bezafibrate on insulin resistance in patients with type 2 diabetes mellitus.

**Methods:**

Twelve Japanese patients with type 2 diabetes mellitus and dyslipidemia (mean age: 59.5 years; fasting plasma glucose: 7.95 mmol/L; hemoglobin A1c [HbA1c]: 7.3%; body mass index: 26.5 kg/m^2^) underwent a glucose clamp and MTT before and after 12 weeks of treatment with 400 mg/day bezafibrate. The glucose infusion rate was measured during the glucose clamp. The patients took a test meal (460 kcal) in the MTT. Plasma glucose and immunoreactive insulin levels were measured at 0 (fasting), 30, 60, 120, and 180 min. Serum C-peptide immunoreactivity, serum lipids, and liver function markers were also measured during the MTT.

**Results:**

Bezafibrate significantly increased the mean glucose infusion rate from 5.78 ± 1.94 to 6.78 ± 2.52 mg/kg/min (p < 0.05). HbA1c improved from 7.30 ± 0.55% to 7.02 ± 0.52% (p < 0.05). In the MTT, fasting plasma glucose decreased from 7.95 ± 1.15 to 6.98 ± 1.07 mmol/L (p < 0.05). The area under the plasma glucose curve from 0 to 180 min decreased significantly from 29.48 ± 5.07 to 27.12 ± 3.98 mmol/h/L (p < 0.05), whereas immunoreactive insulin was unchanged. Furthermore, bezafibrate also significantly improved serum lipids, with decreases in triglyceride levels from 1.84 ± 0.88 to 1.14 ± 0.41 mmol/L (p < 0.05), low-density lipoprotein cholesterol levels from 3.56 ± 0.83 to 2.92 ± 0.55 mmol/L (p < 0.05), and remnant-like particle cholesterol levels decreased from 0.25 ± 0.16 to 0.14 ± 0.06 mmol/L (p < 0.05), and increases in high-density lipoprotein cholesterol levels from 1.50 ± 0.24 to 1.66 ± 0.29 mmol/L (p < 0.05).

**Conclusions:**

Bezafibrate improved glucose intolerance and peripheral insulin resistance in these Japanese patients with type 2 diabetes mellitus and dyslipidemia. Therefore, bezafibrate could be used to treat insulin resistance in patients with type 2 diabetes mellitus and dyslipidemia.

**Trial registration:**

University Hospital Medical Information Network (UMIN) Clinical Trials Registry, UMIN000012462.

## Background

Type 2 diabetes mellitus is a heterogeneous disease characterized by insulin resistance and defective insulin secretion [1]. There are currently two classes of drugs available to treat insulin resistance, thiazolidinediones and metformin; new insulin sensitizers are required. Bezafibrate is currently mainly used to treat hypertriglyceridemia. Bezafibrate was found to reduce the incidence of coronary artery disease, especially non-fatal myocardial infarction, in patients with high baseline triglycerides (≥200 mg/dl) or with metabolic syndrome [2, 3], as well as to reduce the incidence and delay the onset of type 2 diabetes in obese patients [4]. Furthermore, bezafibrate was associated with a lower risk of incident diabetes compared with other fibrates (ciprofibrate, clofibrate, gemfibrozil, and fenofibrate) [5]. In the Japan bezafibrate clinical effectiveness and tolerability (J-BENEFIT) study, a large-scale study of 3316 patients with type 2 diabetes mellitus and dyslipidemia, administration of 200–400 mg/day of bezafibrate for 24 weeks decreased hemoglobin A1c (HbA1c) from 7.69% to 7.22% and fasting blood glucose levels from 9.00 to 7.81 mmol/L [6]. These results indicate that bezafibrate is also effective for the treatment of type 2 diabetes mellitus.

The gold standard for assessing insulin resistance in human is the euglycemic hyperinsulinemic clamp (glucose clamp). In the glucose clamp, most of the infused glucose is taken up by peripheral tissues, primarily muscle [7]. Under the steady-state conditions of euglycemia, the glucose infusion rate (GIR) is a measure of tissue sensitivity to exogenous insulin [8]. A previous study of nondiabetic men with combined hyperlipidemia revealed that bezafibrate did not improve insulin sensitivity assessed by glucose clamps [9]. A study of six patients with type 2 diabetes mellitus also revealed that bezafibrate did not change peripheral insulin sensitivity assessed by glucose clamps [10]. However, that study was small, involving only six patients, and there are no other reports describing the effects of bezafibrate on insulin sensitivity, assessed by glucose clamps, in patients with type 2 diabetes mellitus.

Patients with type 2 diabetes mellitus generally have higher serum triglyceride (TG) and low-density lipoprotein cholesterol (LDL-C) levels and lower high-density lipoprotein cholesterol (HDL-C) levels than people without diabetes [11]. Furthermore, serum TG, LDL-C, and HDL-C levels are worse when HbA1c is high [12]. Intramyocellular lipid levels are inversely associated with insulin sensitivity [13].

Based on these results, we hypothesized that bezafibrate improves glucose intolerance by improving hypertriglyceridemia and skeletal muscle insulin resistance in patients with type 2 diabetes mellitus. To test this hypothesis, we performed glucose clamps and meal tolerance tests (MTT) to examine the effects of bezafibrate on insulin resistance, glucose metabolism, and lipid metabolism in patients with type 2 diabetes mellitus.

## Methods

### Subjects

The subjects were recruited from the outpatient clinic at Tottori University Hospital between January 2009 and August 2012. The entry criteria included patients with type 2 diabetes mellitus and dyslipidemia. Patients with pancreatic disease, liver disease, renal failure, or those taking diabetogenic medications, such as corticosteroids, were excluded from the study. Consecutive subjects previously diagnosed type 2 diabetes mellitus and dyslipidemia were chosen. Type 2 diabetes mellitus was diagnosed based on the criteria of the World Health Organization [14]. Dyslipidemia was defined as follows: TG levels ≥1.7 mmol/L, and/or LDL-C levels ≥3.6 mmol/L, and/or HDL-C levels ≤1.0 mmol/L. Table 1 shows the characteristics and treatments of the patients at baseline. There were five males and seven females. Their mean age was 59.5 years. Two patients were on diet therapy alone and 10 patients were being treated with one or more oral hypoglycemic agents, including sulfonylurea (2 patients), glinides (3), metformin (3), α-glucosidase inhibitors (5), and dipeptidyl peptidase-4 inhibitors (5). None of the patients were treated with a sulfonylurea in combination with a glinide. None of the patients were using thiazolidinediones or insulin. Regarding hypolipidemic agents, statin and ezetimibe were not administered to the same patients.

**Table 1:** **Characteristics and treatments of the subjects at baseline**

Variable	Value
Number	12
Sex (male/female)	5/7
Age (years)	59.5 ± 9.4
Antidiabetic treatments	
Diet alone	2
Oral hypoglycemic agents	10
Sulfonylurea	2
Glinide	3
Metformin	3
α-glucosidase inhibitor	5
Dipeptidyl peptidase-4 inhibitor	5
Thiazolidinedione	0
Insulin	0
Hypolipidemic agents	
Fenofibrate	0
Statin	1
Ezetimibe	4
None	7

Data are shown as the mean ± standard deviation or n.

All of the patients were treated with 400 mg bezafibrate twice daily for 12 weeks. There were no changes in lifestyle interventions or medications during the study period. The patients underwent the glucose clamps and MTTs before and after 12 weeks of treatment with bezafibrate.

HbA1c was measured by high-performance liquid chromatography and was converted to National Glycohemoglobin Standardization Program (NGSP) values using the following officially certified equation: NGSP (%) = 1.02 × Japan Diabetes Society (JDS, %) + 0.25% [15]. The reverse equation is as follows: JDS (%) = 0.980 × NGSP (%) - 0.245%. HbA1c (NGSP) values were also converted to International Federation of Clinical Chemistry values (mmol/mol) using the HbA1c converter developed by Diabetes UK (Macleod House, London, UK).

This study was approved by the Ethical Committee of the Faculty of Medicine, Tottori University. Informed consent was obtained from the patients on the basis of a procedure officially approved by the Ethical Committee.

### Glucose clamp

The glucose clamps were conducted as previously described [16–18]. Briefly, the patients fasted overnight from 21:00 to 09:00 h before each test and visited the clinic in the morning. An antecubital vein was cannulated to administer the infusate, and a dorsal vein was cannulated and kept warm to facilitate venous sampling and provide arterialized venous blood. The glucose clamp was performed to determine insulin sensitivity in peripheral tissue [8] using an artificial endocrine pancreas (STG 22; Nikkiso, Shizuoka, Japan). A primed-constant infusion of insulin (100 mU/m^2^/min) and computer-controlled exogenous infusion of glucose solution were used to achieve steady-state plasma insulin levels and maintain plasma glucose levels at 5.2 mmol/L (95 mg/dL). Using an identical insulin infusion, previous studies have shown that the steady-state plasma insulin level is 1200 pmol/L in patients with type 2 diabetes mellitus [19, 20]. The steady-state GIR was calculated at 90–120 min and the mean GIR during that period was used as a marker of peripheral insulin sensitivity.

### MTT

The MTT was conducted as previously described [16–18] on a different day, generally 2–3 days before the glucose clamp. Briefly, the patients were fasted overnight from 21:00 to 09:00 h before each test. The patients visited the clinic in the morning and consumed a test meal prepared by the Japan Diabetes Society (460 kcal; 15% protein, 35% fat, 50% carbohydrates, and 1.6 g salt) [21]. Plasma glucose and immunoreactive insulin (IRI) were measured at 0 (fasting), 30, 60, 120, and 180 min after the test meal. Serum C-peptide immunoreactivity, TG, and plasma remnant-like particle cholesterol (RLP-C) levels were measured at 0 (fasting) and 120 min. HbA1c, total cholesterol, LDL-C, HDL-C, high-sensitivity C-reactive protein, adiponectin, aspartate aminotransferase, alanine aminotransferase (ALT), and γ-glutamyltranspeptidase isozyme (γ-GTP) levels were measured using fasting samples. Plasma glucose levels were measured using the glucose oxidase method. Plasma insulin and C-peptide immunoreactivity levels were measured using chemiluminescent immunoassays (human insulin and C-peptide immunoreactivity CLIA kit; Kyowa Medex, Tokyo, Japan). Plasma insulin was defined as IRI. Plasma adiponectin levels were measured using an enzyme-linked immunosorbent assay (human adiponectin ELISA kit; Otsuka, Tokyo, Japan). RLP-C levels were measured using a homogeneous assay (MetaboLead RemL-C kit: Kyowa Medex). High-sensitive C-reactive protein was measured by nephelometry (N-Latex CRP II kit; Siemens Healthcare Diagnostics, Tokyo, Japan).

### Statistical analysis

Data are expressed as means ± standard error of the mean. Baseline data were compared with those obtained after treatment using paired *t* tests. Statistical significance was defined as p < 0.05. SPSS software version 15.0 (SPSS, Chicago, IL, USA) was used for statistical analyses.

## Results

**Figure 1:**
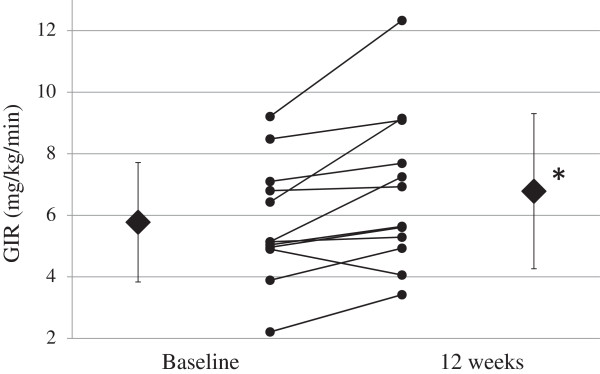
**Individual and mean changes in GIR at baseline and after 12 weeks of treatment with bezafibrate. ♦** = mean GIR; ● = individual GIR (n = 12). GIR = glucose infusion rate. *p < 0.05 vs. baseline (paired *t* test).

Figure 1 shows the changes in the steady-state mean GIR in the glucose clamps performed before and after 12 weeks of treatment with bezafibrate. The GIR significantly increased from 5.78 mg/kg/min before bezafibrate treatment to 6.78 mg/kg/min after treatment (p < 0.05). The mean plasma IRI level was 1380 ± 538 pmol/L at steady-state during the glucose clamp.

Table 2 shows the clinical characteristics of the subjects at baseline and after 12 weeks of treatment with bezafibrate. HbA1c (from 7.30% to 7.02%) and fasting plasma glucose (FPG) levels (from 7.95 to 6.98 mmol/L) decreased significantly during this treatment period (p < 0.05). The homeostatic model assessment of insulin resistance (HOMA-IR) tended to improve (from 2.66 to 2.20, p = 0.08). Regarding lipid levels, TG (p < 0.01), RLP-C (p < 0.05), total cholesterol (p < 0.05), and LDL-C (p < 0.05) levels decreased significantly and HDL-C (p < 0.05) levels increased significantly between baseline and 12 weeks of treatment. Regarding liver function markers, ALT (p < 0.01) and γ-GTP levels (p < 0.05) decreased significantly after treatment. Body mass index and waist circumference did not increase after bezafibrate treatment.

**Figure 2:**
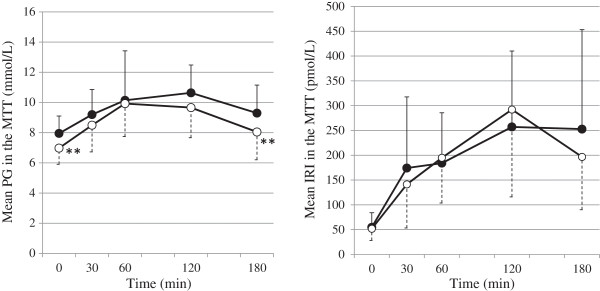
**Plasma glucose levels and IRI in the MTTs before and after 12 weeks of treatment with bezafibrate.** ● = mean values at baseline; ○ = mean values after 12 weeks of treatment with bezafibrate (n = 12). IRI = immunoreactive insulin; MTT = meal tolerance test; PG = plasma glucose. **p < 0.01 vs. baseline (paired *t* test).

**Figure 3:**
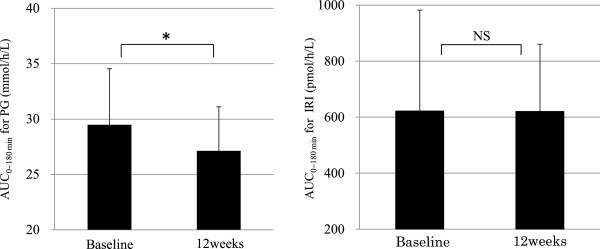
**Areas under the plasma glucose and IRI curves from 0 to 180 min in the MTT at baseline and after 12 weeks of treatment with bezafibrate (n = 12).** AUC_0–180 min_ = area under the curve from 0 to 180 min; IRI = immunoreactive insulin; PG = plasma glucose. *p < 0.05 vs. baseline (paired *t* test).

Figures 2 and 3 show the changes in plasma glucose and IRI levels in the MTTs performed before and after 12 weeks of treatment with bezafibrate. In addition to a decrease in FPG, plasma glucose levels at 180 min after the meal decreased significantly from 9.30 ± 1.85 to 8.05 ± 1.84 mmol/L (p < 0.01). The area under the plasma glucose curve from 0 to 180 min decreased significantly from 29.5 ± 5.1 to 27.1 ± 4.0 mmol/h/L (p < 0.05). There were no significant changes in IRI, including the area under the IRI curve from 0 to 180 min.

**Table 2:** **Results of the MTTs performed before and after 12 weeks of treatment with bezafibrate**

		Baseline	12 weeks	p
HbA1c (NGSP) (%)		7.30 ± 0.55	7.02 ± 0.52	<0.05
HbA1c (NGSP) (mmol/mol)		54.5 ± 5.6	51.6 ± 5.3	<0.05
FPG (mmol/L)		7.95 ± 1.15	6.98 ± 1.07	<0.01
HOMA-IR		2.66 ± 1.42	2.20 ± 0.94	0.08
HOMA-β		38.97 ± 34.28	46.44 ± 28.59	0.12
Matsuda index		5.59 ± 2.04	5.98 ± 2.48	0.47
Insulinogenic index		0.92 ± 0.81	0.67 ± 0.69	0.18
IRI (pmol/L)	Fasting	54.7 ± 29.4	51.8 ± 23.5	0.51
	120 min*	257.1 ± 153.0	292.2 ± 176.1	0.18
CPR (nmol/L)	Fasting	0.55 ± 0.25	0.50 ± 0.14	0.28
	120 min*	1.54 ± 0.52	1.56 ± 0.42	0.82
TG (mmol/L)	Fasting	1.84 ± 0.88	1.14 ± 0.41	<0.01
	120 min*	2.13 ± 0.97	1.47 ± 0.40	<0.01
RLP-C (mmol/L)	Fasting	0.25 ± 0.16	0.12 ± 0.05	<0.05
	120 min*	0.26 ± 0.16	0.14 ± 0.06	<0.05
TC (mmol/L)		5.83 ± 1.03	5.30 ± 0.60	<0.05
LDL-C (mmol/L)		3.56 ± 0.83	2.92 ± 0.55	<0.05
HDL-C (mmol/L)		1.50 ± 0.24	1.66 ± 0.29	<0.05
hs-CRP (mg/dl)		0.11 ± 0.10	0.09 ± 0.12	0.38
Adiponectin (μg/mL)		7.81 ± 2.52	8.37 ± 3.90	0.31
AST (IU/L)		32.3 ± 21.7	28.9 ± 19.7	0.05
ALT (IU/L)		39.8 ± 25.8	28.8 ± 19.6	<0.01
γ-GTP (IU/L)		66.5 ± 56.2	30.7 ± 18.2	<0.05
BMI (kg/m^2^)		26.5 ± 3.1	26.2 ± 2.9	0.14
Waist circumference (cm)		91.8 ± 7.6	93.5 ± 9.2	0.14

Data are mean ± standard deviation.

TC, LDL-C, HDL-C, RLP-C, hs-CRP, adiponectin, AST, ALT, and γ-GTP were measured using fasting blood samples.

*Measured at 120 min after the test meal.

HbA1c = hemoglobin A1c; NGSP = National Glycohemoglobin Standarization Program; FPG = fasting plasma glucose; HOMA-IR = homeostatic model assessment of insulin resistance; HOMA-β = homeostasis model assessment of β cell function; IRI = immunoreactive insulin; CPR = C-peptide immunoreactivity; TG = triglycerides; RLP-C = remnant-like particle cholesterol; LDL-C = low-density lipoprotein cholesterol; HDL-C = high-density lipoprotein cholesterol; hs-CRP = high-sensitivity C-reactive protein; AST = aspartate aminotransferase; ALT = alanine aminotransferase; γ-GTP = γ-glutamyltranspeptidase; BMI = body mass index.

## Discussion

In this study, bezafibrate improved the GIR by 17% (from 5.78 to 6.78 mg/kg/min) in glucose clamps in patients with type 2 diabetes mellitus and dyslipidemia. Bezafibrate also improved glucose intolerance, as shown by decreases in HbA1c and FPG, similar to the J-BENEFIT study [6]. In the steady-state euglycemic condition, the GIR is indicative of skeletal muscle insulin resistance. Our results indicate that bezafibrate improved skeletal muscle insulin resistance without increasing insulin secretion.

HOMA-IR, which was developed by Turner et al., is an index of insulin resistance, predominantly hepatic insulin resistance [22]. A previous study of 168 patients with type 2 diabetes mellitus revealed that HOMA-IR and insulin were not altered by bezafibrate [23]. In the J-BENEFIT study [6], bezafibrate improved (i.e. decreased) HOMA-IR from 4.46 to 3.38 in patients with type 2 diabetes mellitus and dyslipidemia. Another study showed that bezafibrate attenuated the progression of insulin resistance by evaluating HOMA-IR in patients with coronary artery disease [24]. In our study, bezafibrate significantly decreased liver function markers, especially ALT and γ-GTP. HOMA-IR tended to improve and IRI did not change after 12 weeks of treatment compared with before treatment. These results suggest that bezafibrate improved insulin resistance in the liver, and in skeletal muscle without increasing insulin secretion.

Peroxisome proliferator-activated receptor subtypes (PPARs) are members of the nuclear receptor superfamily [25]. There are three PPARs; PPARα, PPRAδ, and PPARγ [26]. PPARα is highly expressed in hepatocytes, cardiomyocytes, enterocytes and the proximal tubule cells of kidney. PPARδ is highly expressed in skeletal muscle, and PPARγ is mainly expressed in adipose tissue, the immune system, and the large intestine [27–29]. Of note, PPARγ plays important roles as a regulator of glucose and lipid homeostasis [26].

Fenofibrate is another fibrate used to treat hypertriglyceridemia. The large-scale Fenofibrate Intervention and Event Lowering in Diabetes (FIELD) study revealed that fenofibrate did not improve HbA1c in patients with type 2 diabetes mellitus and hyperlipidemia [30]. Fenofibrate is a PPARα-specific agonist, whereas bezafibrate is a pan-PPAR agonist that activates all three PPAR isoforms [31]. It was reported that activation of all three isoforms is better than activation of a single isoform in terms of improving insulin resistance [32]. Bezafribrate is also associated with weaker mitochondrial toxicity than fenofibrate [33]. Plasma fatty acids are an important metabolic substrate in skeletal muscle [34] and skeletal muscle is responsible for approximately 80% of insulin-stimulated glucose uptake [29]. Bezafibrate normalizes the fatty acid composition of skeletal muscle, which is closely related to insulin resistance [35]. Intramyocellular lipid levels are inversely associated with insulin sensitivity [13], and an increase in skeletal muscle TG content due to hypertriglyceridemia exacerbates insulin resistance. Therefore, by activating PPARα, PPARδ, and PPARγ together and reducing TG in skeletal muscles, bezafibrate might improve glucose intolerance and insulin resistance in skeletal muscle.

Bezafibrate was reported to improve fatty liver and non-alcoholic fatty liver disease in patients with type 2 diabetes [36]. The dual PPARα and PPARδ agonist GFT505 was reported to suppress endogenous glucose production and improve hepatic insulin sensitivity [37]. An improvement in fatty liver and co-activation of PPARα and PPARδ is expected to contribute to the improvement in hepatic insulin sensitivity. However, to accurately measure hepatic insulin sensitivity, it is necessary to use a radioisotope [38, 39]. Unfortunately, it is difficult to perform studies using radioisotopes in subjects at our institute. Further studies are necessary to examine the direct effects of bezafibrate on hepatic insulin sensitivity.

There are two classes of drugs available to treat insulin resistance, thiazolidinediones and metformin. Prior studies have reported the effects of treatment with a thiazolidinedione or metformin on the GIR in glucose clamps. For example, one study showed that 12 weeks of treatment with pioglitazone increased the GIR from 8.2 to 9.2 mg/kg/min in patients with type 2 diabetes mellitus [19]. Similarly, 3 months of treatment with low-dose metformin (750 mg/day) increased the GIR from 6.24 to 7.82 mg/kg/min in obese Japanese patients with type 2 diabetes mellitus [40]. In our study, bezafibrate increased the GIR from 5.78 to 6.78 mg/kg/min, and had similar effects to pioglitazone and metformin in terms of improving peripheral insulin resistance. Moreover, bezafibrate did not result in weight gain, unlike pioglitazone. Furthermore, in the present study, three patients were administered 750 mg/day or 500 mg/day metformin. In these three patients, bezafibrate increased the GIR from 5.15 to 7.11 mg/kg/min while continuing metformin. These results indicate that bezafibrate has additive effects on improving insulin sensitivity in metformin-treated patients. Meanwhile, some adverse effects of metformin and thiazolidinediones have been reported. Metformin is associated with gastrointestinal symptoms, such as diarrhea or nausea. Lactic acidosis may also occur albeit rarely in metformin-treated patients. Thiazolidinedione, as PPARγ-specific agonists, can cause water retention, weight gain, peripheral edema, and congestive heart failure. Selective overexpression of a constitutively active form of PPARδ in mouse adipose tissue induces significant weight loss and protects against high-fat diet-induced obesity and dyslipidemia [41]. As a pan-PPAR agonist, bezafibrate can simultaneously improve insulin sensitivity by activating PPARγ and attenuate weight gain by activating PPARδ [42]. By activating PPARδ, it is thought that bezafibrate therapy does not induce weight gain.

Some large-scale studies have also examined the safety of bezafibrate. For example, in the J-BENEFIT study, adverse drug reactions occurred in 5.1% of bezafibrate-treated patients, with increased blood creatine phosphokinase (0.8%), blood creatinine (0.8%), blood urea (0.5%), renal impairment (0.3%) and asparate aminotransferase (0.3%) being the most common events [6]. Similarly, in the Japanese safety and efficacy of long-term combination therapy with bezafibrate and ezetimibe in patients with dyslipidemia study (J-COMPATIBLE), adverse drug reactions occurred in 6.4% of patients treated with the combination of bezefibrate and ezetimibe [43]. In our small study involving 12 patients, there were no adverse drug reactions during treatment with bezafibrate.

Our study has several limitations, including the small number of patients, the lack of a control group, and the use of various oral hypoglycemic agents in combination with bezafibrate during the 12-week treatment period. Because only 12 subjects participated in this study, our results require confirmation in a larger study. The effect of bezafibrate on insulin secretion was assessed by calculating Matsuda and insulinogenic indices from MTTs. Both indices were not significantly affected, suggesting that bezafibrate does not alter insulin secretion. Indeed, bezafibrate did not change IRI levels in the MTTs from 0 to 180 min. However, because the Matsuda and insulinogenic indices were originally derived from the results of OGTTs, they may not be appropriate for assessing insulin resistance and acute insulin secretion during MTTs. Despite these limitations, we consider that our findings indicate that bezafibrate improves insulin resistance.

## Conclusions

Bezafibrate improves glucose intolerance in patients with type 2 diabetes mellitus and dyslipidemia. The increase in the GIR in glucose clamps indicates that bezafibrate improves peripheral insulin resistance, particularly in skeletal muscle. These results suggest that bezafibrate could be used to treat insulin resistance in patients with type 2 diabetes mellitus and dyslipidemia.

## Abbreviations

ALT: Alanine aminotransferase
FPG: Fasting plasma glucose
GIR: Glucose infusion rate
HbA1c: Hemoglobin A1c
HDL-C: High-density lipoprotein cholesterol
HOMA-β: Homeostasis model assessment beta
HOMA-IR: Homeostatic model assessment of insulin resistance
IRI: Immunoreactive insulin
J-BENEFIT: Japan bezafibrate clinical effectiveness and tolerability study
JDS: Japan Diabetes Society
LDL-C: Low-density lipoprotein cholesterol
MTT: Meal tolerance test
NGSP: National glycohemoglobin standardization program
PPAR: Peroxisome proliferator-activated receptor
RLP-C: Remnant-like particle cholesterol
TG: Triglycerides.
